# Supplementary material for: End Sequence Analysis Toolkit (ESAT) expands the extractable information from single-cell RNA-seq data
Source: Genome Res. 2016 Oct;26(10):1397–410. doi: 10.1101/gr.207902.116 (PMC5052061; doi:10.1101/gr.207902.116)
Supplement: Supplemental Material [file supp_gr.207902.116_Supp_Materials.tar.gz › ESAT-esat_v0.1/doc/html/classbroad_1_1core_1_1motif_1_1meme_1_1_a_m_e_result_reader.html]

ESAT: broad.core.motif.meme.AMEResultReader Class Reference


ESAT


- Main Page
- Related Pages
- Packages
- Classes
- Files

- Class List
- Class Index
- Class Hierarchy
- Class Members

All Classes Namespaces Files Functions Variables Enumerator Pages

- broad
- core
- motif
- meme
- AMEResultReader

Classes |
Public Member Functions |
List of all members

broad.core.motif.meme.AMEResultReader Class Reference

|  |  |
| --- | --- |
| Classes | |
| class | **AMEMatch** |
|  | |

|  |  |
| --- | --- |
| Public Member Functions | |
|  | AMEResultReader (BufferedReader br) throws IOException |
|  | |
|  | AMEResultReader (InputStream is) throws IOException |
|  | |
|  | AMEResultReader (String inputFile) throws IOException |
|  | |
|  | AMEResultReader (File inputFile) throws IOException |
|  | |

## Constructor & Destructor Documentation

|  |  |  |  |  |  |
| --- | --- | --- | --- | --- | --- |
| broad.core.motif.meme.AMEResultReader.AMEResultReader | ( | BufferedReader | *br* | ) | throws IOException |

|  |  |  |  |  |  |
| --- | --- | --- | --- | --- | --- |
| broad.core.motif.meme.AMEResultReader.AMEResultReader | ( | InputStream | *is* | ) | throws IOException |

|  |  |  |  |  |  |
| --- | --- | --- | --- | --- | --- |
| broad.core.motif.meme.AMEResultReader.AMEResultReader | ( | String | *inputFile* | ) | throws IOException |

|  |  |  |  |  |  |
| --- | --- | --- | --- | --- | --- |
| broad.core.motif.meme.AMEResultReader.AMEResultReader | ( | File | *inputFile* | ) | throws IOException |

---

The documentation for this class was generated from the following file:

- java/broad/core/motif/meme/AMEResultReader.java


---

Generated on Fri Aug 15 2014 15:10:54 for ESAT by  

 1.8.7
